# Performance Comparison of Bio-Inspired Algorithms for Optimizing an ANN-Based MPPT Forecast for PV Systems

**DOI:** 10.3390/biomimetics9100649

**Published:** 2024-10-21

**Authors:** Rafael Rojas-Galván, José R. García-Martínez, Edson E. Cruz-Miguel, José M. Álvarez-Alvarado, Juvenal Rodríguez-Resendiz

**Affiliations:** 1 Facultad de Ingeniería, Universidad Autónoma de Querétaro, Santiago de Queretaro 76010, Mexicojuvenal@uaq.edu.mx (J.R.-R.); 2 Faculty of Electronics and Communications Engineering, Universidad Veracruzana, Poza Rica 93390, Mexico; edsoncruz@uv.mx

**Keywords:** MPPT, ANN, optimization algorithm, bioinspired, GWO, PSO, CS, SSA

## Abstract

This study compares bio-inspired optimization algorithms for enhancing an ANN-based Maximum Power Point Tracking (MPPT) forecast system under partial shading conditions in photovoltaic systems. Four algorithms—grey wolf optimizer (GWO), particle swarm optimization (PSO), squirrel search algorithm (SSA), and cuckoo search (CS)—were evaluated, with the dataset augmented by perturbations to simulate shading. The standard ANN performed poorly, with 64 neurons in Layer 1 and 32 in Layer 2 (MSE of 159.9437, MAE of 8.0781). Among the optimized approaches, GWO, with 66 neurons in Layer 1 and 100 in Layer 2, achieved the best prediction accuracy (MSE of 11.9487, MAE of 2.4552) and was computationally efficient (execution time of 1198.99 s). PSO, using 98 neurons in Layer 1 and 100 in Layer 2, minimized MAE (2.1679) but had a slightly longer execution time (1417.80 s). SSA, with the same neuron count as GWO, also performed well (MSE 12.1500, MAE 2.7003) and was the fastest (987.45 s). CS, with 84 neurons in Layer 1 and 74 in Layer 2, was less reliable (MSE 33.7767, MAE 3.8547) and slower (1904.01 s). GWO proved to be the best overall, balancing accuracy and speed. Future real-world applications of this methodology include improving energy efficiency in solar farms under variable weather conditions and optimizing the performance of residential solar panels to reduce energy costs. Further optimization developments could address more complex and larger-scale datasets in real-time, such as integrating renewable energy sources into smart grid systems for better energy distribution.

## 1. Introduction

Advancements in modern technology have made renewable energy a highly discussed global topic driven by the ongoing energy crisis. As a result, the quest for green energy has introduced various renewable sources, including solar, wind, ocean, hydropower, biomass, geothermal resources, biofuels, and hydrogen, which are derived directly from the sun or geothermal heat. Solar energy, in particular, is abundant and environmentally safe, making it a reliable power source [1].

Engineers developing solar inverters use Maximum Power Point Tracking (MPPT) algorithms to optimize the power output of photovoltaic (PV) solar systems. These algorithms adjust for changes in irradiance and temperature to ensure that the photovoltaic system consistently produces maximum power [2]. MPPT algorithms are critical because they address these variable factors to maintain optimal performance [3,4,5,6]. MPPT algorithms can be classified according to their voltage, current, or duty cycle control variable. Their primary advantage lies in their ability to solve non-linear problems and generate optimal solutions efficiently, including multiple peaks for global maxima. These methods outperform conventional algorithms in tracking performance [7,8].

The review of previous research on MPPT algorithms initially points to the Perturb and Observe (P&O) method, recognized for its simplicity and fundamental nature but often unsuitable due to its lower accuracy [9,10]. The system is expected to oscillate around the Maximum Power Point (MPP) in real-world scenarios, implying that a continuous perturbation in a fixed direction can significantly move the operating point away from the true MPP. This behavior persists until the irradiance is decreased or eliminated. Among the alternative techniques available, the IC method is one of the most prominent [11]. Although the method offers higher accuracy than the P&O method, its implementation is complex. Both Perturb and Observe and Incremental Conductance are “slope climbing” algorithms that allow for locating the point of maximum local power on the power curve under the operating conditions of a solar PV system. However, the Fractional Open Circuit Voltage (FOCV) and Fractional Short Circuit Current (FSCC) methods are also widely used, as they are more efficient than the P&O method [12].

The main objective of PV systems is to extract as much solar energy as possible quickly and reliably, even under variable environmental conditions, using MPPT techniques. Under uniform solar irradiance, many of these techniques achieve satisfactory system performance. However, when Partial Shade Condition (PSC) occurs, the MPPT process becomes more complicated as the characteristics of the photovoltaic system become non-linear. This can lead to a Local Maximum Power Point (LMPP), even though there is only one Global Maximum Power Point (GMPP). In addition, PSCs can cause power losses, the formation of hot spots, and risks to the safety and reliability of the system. Several methods have been designed to overcome these obstacles that allow MPPT to be performed efficiently under PSC.

The research of MPPT techniques under uniform and non-uniform irradiances has been a significant focus. Recently, there has been a growing interest in metaheuristic techniques, often used in machine learning applications and now being explored for optimization in commercial settings.

This article focuses on the application of metaheuristic algorithms in the MPPT approach, providing key contributions:

- Algorithm types: The study explores bio-stimulated, nature-inspired, and swarm-based algorithms, including particle swarm optimization (PSO), grey wolf optimizer (GWO), squirrel search algorithm (SSA), and cuckoo search (CS), highlighting their distinct approaches to optimization.
- Methodology: To compare these algorithms, a partial shade condition is introduced to the base dataset, simulating real-world challenges in photovoltaic systems.
- Neural network optimization: The optimization algorithms are also tasked with tuning the number of neurons in each layer of the ANN, allowing them to propose alternative network architectures that minimize MAE, MSE, and $R^{2}$.

This paper is organized as follows. Section 2 discusses the state-of-the-art and theoretical background, where the relevant information is analyzed. Section 3 outlines the methodology and the approach used. Section 4 and Section 5 present the results and the discussion, respectively. Finally, the conclusions are summarized in Section 6.

## 2. State of the Art

**Figure 1:**
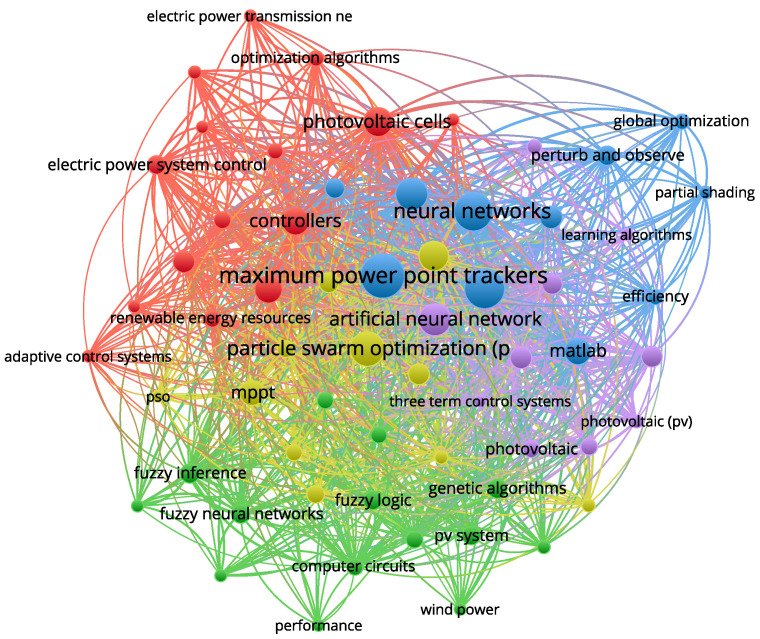
Bibliometric network using keywords such as MPPT, ANN, and optimization algorithm in Scopus.

The bibliometric network shown in Figure 1 highlights critical research trends in the field of MPPT for photovoltaic systems. It strongly focuses on integrating neural networks and optimization algorithms, such as PSO with MPPT techniques. Significant research also links photovoltaic technology with control systems and efficiency improvements.

The clusters suggest that interdisciplinary approaches combining artificial intelligence, optimization, and control methods are key to advancing MPPT and photovoltaic system performance. This indicates a trend towards more sophisticated AI-driven techniques to enhance the efficiency and reliability of renewable energy systems.

The state-of-the-art analysis reveals that PSO is the predominant algorithm employed for MPPT in photovoltaic systems. The popularity of PSO stems from its consistent and reliable performance in various conditions, including partial shading and dynamic weather changes. Its effectiveness is often bolstered by careful tuning of hyperparameters, such as inertia weight and cognitive coefficients, which optimize power output and efficiency.

However, the extensive reliance on PSO also highlights the need to explore alternative optimization algorithms that could offer superior performance in specific scenarios. While PSO excels in general applications, algorithms like GWO have shown the potential to achieve faster convergence times or better accuracy under challenging conditions, such as rapidly changing environments.

Moreover, recent developments in optimization algorithms, such as SSA and CS, present promising alternatives that could enhance the speed of MPPT systems. These newer algorithms are designed to tackle complex optimization problems, potentially offering advantages over more established methods, such as PSO.

This analysis shown in Table 1 underscores the relevance of investigating these alternatives, as they could outperform PSO in areas with limitations, such as speed or adaptability to sudden operating conditions. Pursuing such options could lead to developing more efficient and responsive MPPT systems, ultimately enhancing the overall stability and efficiency of photovoltaic energy systems.

### 2.1. Artificial Neural Network (ANN) Model

The ANN used is designed to predict the generated power (*P*) based on various independent variables, such as temperature, irradiance, voltage at maximum power (Vmp), and current at maximum power (Imp). The ANN can be described by the following model.

#### 2.1.1. Structure of the ANN

The ANN model consists of:

- An input layer with neurons corresponding to the number of input features.
- One or more hidden layers with a specified number of neurons.
- An output layer with a single neuron representing the predicted power.

Input Layer

The input layer receives the normalized input features, as shown in Equation (1):

(1) X=[Temperature,Irradiance,Vmp,Imp]

where *X* represents the key variables, such as temperature, irradiance, the voltage at maximum power (Vmp), and the current at maximum power (Imp).

Each hidden layer applies a transformation to the inputs as it follows.

Weighted Sum: For each neuron *j* in the hidden layer *l*, the weighted sum is computed as shown in Equation (2):

(2) $$z_{j}^{l}=\sum_{i=1}^{n_{l}}w_{ij}^{l}a_{i}^{l-1}+b_{j}^{l}$$

where:

- $w_{ij}^{l}$ are the weights connecting neuron *i* from the previous layer (l−1) to neuron *j* in the current layer *l*.
- $a_{i}^{l-1}$ are the activations from the previous layer (l−1).
- $b_{j}^{l}$ is the bias term for neuron *j* in layer *l*.

#### 2.1.2. Activation Function

An activation function σ is applied to the weighted sum to introduce non-linearity, as shown in Equation (3):

(3) $$a_{j}^{l}=\sigma \left(z_{j}^{l}\right)$$

where $a_{j}^{l}$ is the activation of neuron *j* in layer *l*, and $z_{j}^{l}$ is the weighted sum as computed in Equation (2).

Common activation functions include ReLU (Rectified Linear Unit), sigmoid, and tanh.

#### 2.1.3. Output Layer

The output layer produces the final prediction *P* as shown in Equation (4):

(4) $$P=a_{1}^{L}$$

where *L* is the last layer (output layer) and $a_{1}^{L}$ is the activation of the single output neuron.

#### 2.1.4. ANN Equation

The ANN prediction can be expressed as a composite function of the input features as shown in Function (5).

(5) $$P=\;\sigma \left(w_{1}^{L}\left(\sigma \left(w_{11}^{L-1}\left(\cdots \sigma \left(w_{11}^{1}X_{1}+w_{12}^{1}X_{2}+w_{13}^{1}X_{3}+w_{14}^{1}X_{4}+b_{1}^{1}\right)+\cdots \right)+b_{1}^{L-1}\right)\right)+b_{1}^{L}\right)$$

Here, $X_{1},X_{2},X_{3},$ and $X_{4}$ represent the input features (Temperature, Irradiance, Vmp, Imp), *w* and *b* represent the weights and biases, and σ represents the activation functions applied at each layer.

#### 2.1.5. Incorporation of Data into the ANN Model

The input features (Temperature, Irradiance, Vmp, Imp) are fed into the input layer of the ANN. Each input feature is multiplied by its corresponding weights and added to the bias terms as it passes through each layer. The activation function transforms these weighted sums to produce activations for the next layer. This process continues through the hidden layers until the final prediction is generated at the output layer.

In this model, the network learns the optimal weights and biases during training by minimizing the error between the predicted and actual power in the dataset. This optimization is typically performed using algorithms such as gradient descent, often implemented as the Adam optimizer.

The training process adjusts the weights and biases iteratively to improve the accuracy of the predictions, allowing the ANN to effectively model the complex relationships between the input features and the power output.

### 2.2. Particle Swarm Optimization

PSO is a nature-inspired optimization algorithm based on the social behavior of birds flocking or fish schooling [20]. This algorithm models the collaborative process of individuals (particles) as they explore the search space to find the optimal solution [21]. The key components of PSO are:

- Social Sharing of Information: In the PSO algorithm, each particle modifies its position in the search space based on its individual experience (personal best position) as well as the collective experience of the swarm (global best position). This collaborative approach facilitates the swarm to approach optimal solutions.
- Velocity and position update: The algorithm adjusts the position of each particle based on its velocity. This velocity is determined by considering the particle’s previous velocity, the distance to its personal best position, and the distance to the best global position discovered so far.
- Balance Between Exploration and Exploitation: PSO balances exploration (searching new areas of the search space) and exploitation (refining known reasonable solutions) through parameters such as inertia weight, cognitive coefficient, and social coefficient.

The position and velocity update rules for the particles can be represented mathematically. The velocity update, Equation (6), is:

(6) $${\vec{v}}_{i}^{t+1}=w\cdot {\vec{v}}_{i}^{t}+c_{1}\cdot r_{1}\cdot \left({\vec{p}}_{i}^{t}-{\vec{x}}_{i}^{t}\right)+c_{2}\cdot r_{2}\cdot \left({\vec{g}}^{t}-{\vec{x}}_{i}^{t}\right)$$

where:

- ${\vec{v}}_{i}^{t}$ is the velocity of particle *i* at iteration *t*,
- *w* is the inertia weight controlling the influence of the previous velocity,
- $c_{1}$ is the cognitive coefficient representing the influence of the particle’s best position,
- $c_{2}$ is the social coefficient representing the influence of the global best position,
- $r_{1}$ and $r_{2}$ are random numbers uniformly distributed in the range [0,1],
- ${\vec{p}}_{i}^{t}$ is the personal best position of particle *i* at iteration *t*,
- ${\vec{g}}^{t}$ is the global best position the swarm finds at iteration *t*.

After updating the velocity, the position of each particle is updated using Equation (7):

(7) $${\vec{x}}_{i}^{t+1}={\vec{x}}_{i}^{t}+{\vec{v}}_{i}^{t+1}$$

where:

- ${\vec{x}}_{i}^{t}$ is the position of particle *i* at iteration *t*,
- ${\vec{v}}_{i}^{t+1}$ is the updated velocity of particle *i* for the next iteration.

By iteratively updating the velocities and positions of the particles, PSO converges to an optimal solution by leveraging both individual learning (personal best) and social learning (global best), effectively balancing the exploration of the search space and the exploitation of known good solutions.

### 2.3. Gray Wolf Optimizer

In 2014, a new algorithm known as GWO was introduced, which joined the family of swarm intelligence-based optimization methods, as shown in [13]. The hunting behavior of gray wolves inspires the GWO algorithm. These animals hunt in packs and operate under a four-tier hierarchy. The pack leaders, alpha wolves (α), make all hunting-related decisions. Betas (β) are subleaders who assist the alphas in making decisions. Next in the hierarchy are deltas (δ), obedient to alphas and betas but rank higher than omegas (ω). At the lowest level, omegas shows deference to all other wolves of higher rank [22].

The GWO approach divides candidate solutions into four groups, with alpha being the best, beta being the second best, and delta being the third best, to mimic the leadership hierarchy. Omega refers to the remaining solutions. When hunting, grey wolves encircle their prey, and this behavior can be modeled using Equations (8) and (9):

(8) $$\vec{D}=\left|\vec{C}\cdot {\vec{x}}_{p}\left(t\right)-\vec{x}\left(t\right)\right|$$

(9) $$\vec{x}\left(t+1\right)=\left|{\vec{x}}_{p}\left(t\right)-\vec{A}\cdot \vec{D}\right|$$

where *t* denotes the current iteration, $\vec{A}$ and $\vec{C}$ are coefficient vectors, ${\vec{x}}_{p}$ is the position vector of the prey, and $\vec{x}$ is the position vector of the grey wolf. Calculations for the vectors $\vec{A}$ and $\vec{C}$ are shown in Equations (10) and (11):

(10) $$\vec{A}=2\vec{a}\cdot {\vec{r}}_{1}-\vec{a}$$

(11) $$\vec{C}=2{\vec{r}}_{2}$$

where ${\vec{r}}_{1}$ and ${\vec{r}}_{2}$ are random numbers in the range [0, 1], and the elements of $\vec{a}$ are linearly decreased from 2 to 0 throughout iterations. Beta and delta wolves occasionally participate in the hunt, but leadership and control primarily rest with the alpha wolf. The delta and omega wolves care for wounded wolves within the pack. Because of this, the alpha is considered the most reliable source for determining the location of prey. Once the target stops, the gray wolves complete the hunt by attacking it.

### 2.4. Cuckoo Search Algorithm

The CS algorithm was first introduced by Yang and Deb in 2009 [23], inspired by the breeding behavior of cuckoos. The CS algorithm follows three main rules:

- Each cuckoo lays one egg at a time and places it in a randomly chosen nest.
- The best nests with high-quality solutions will carry over to the next generation.
- The number of host nests is fixed, and there is a probability $P_{a}\in \left[0,1\right]$ that a host bird will discover an alien egg.

The following Levy flight Equation (12) describes the position update of the CS:

(12) $$x_{i}^{t+1}=x_{i}^{t}+\alpha ⊕\mathrm{Levy}\left(\lambda \right)$$

where $X_{i}=\left[x_{1},x_{2},x_{3},\ldots ,x_{D}\right]$ represents a solution vector, *D* is the problem dimensionality, and λ>0 represents the step-size scale. The term *t* denotes the iteration number. The product symbol ⊕ denotes entrywise multiplication, and Levy(λ) generates a random walk with step lengths drawn from a Lévy distribution, as shown in Equation (13):

(13) $$\mathrm{Levy}\left(\lambda \right)\approx t^{-\lambda },\;\left(1<\lambda \leq 3\right)$$

### 2.5. Squirrel Search Algorithm

The SSA is a nature-inspired optimization algorithm based on the foraging behavior of squirrels [24]. This algorithm models the dynamic search process of squirrels as they gather food and store it in different locations to survive during winter. The key components of SSA are:

- Foraging Strategy: Squirrels use a dynamic foraging strategy that includes searching for food, hoarding it, and retrieving it later. This strategy helps squirrels to adapt to seasonal changes and ensures their survival.
- Energy and Food Storage: The algorithm considers the energy levels of squirrels and the amount of food stored. Squirrels with higher energy levels can search more extensively, while those with lower energy levels focus on retrieving stored food.
- Predator Avoidance: Squirrels need to avoid predators while foraging. The SSA models this by incorporating a risk factor that influences the foraging paths of the squirrels.

The position update rules for the squirrels can be represented mathematically. If a squirrel finds a high-quality food source, it updates its position towards that source. The position update Equation (14) is:

(14) $${\vec{x}}_{i}^{t+1}={\vec{x}}_{i}^{t}+\beta \cdot \left({\vec{x}}_{best}^{t}-{\vec{x}}_{i}^{t}\right)+\gamma \cdot \left({\vec{x}}_{rand}^{t}-{\vec{x}}_{i}^{t}\right)$$

where:

- ${\vec{x}}_{i}^{t}$ is the position of squirrel *i* at iteration *t*,
- ${\vec{x}}_{best}^{t}$ is the position of the best food source found so far,
- ${\vec{x}}_{rand}^{t}$ is the position of a randomly selected food source,
- β and γ are coefficients controlling the influence of the best and random food sources, respectively.

A risk factor R∈[0,1] modulates the risk of predator encounters. When a squirrel encounters a predator, it updates its position using Equation (15):

(15) $${\vec{x}}_{i}^{t+1}={\vec{x}}_{i}^{t}+\delta \cdot \vec{R}$$

where:

- δ is a coefficient representing the impact of the predator risk,
- $\vec{R}$ is a random vector indicating the direction of escape.

By iteratively updating the position of the squirrels, the SSA converges on an optimal solution that balances exploration and exploitation, adapts to environmental changes, and avoids risks.

### 2.6. Performance Metrics

When developing an artificial intelligence model, it is crucial to assess the model’s quality. This assessment involves determining how effectively the model has been trained using the training data and how accurately it moves test observations [25].

Solar energy monitoring systems require the ability to analyze complex atmospheric datasets. To address this, optimization algorithms selectively filter the necessary information. This process involves removing irrelevant features to prevent model performance degradation [26]. To evaluate the effectiveness of the model, various metrics are employed, including the following.

- $R^{2}$: This metric determines what fraction of the variation in the dependent variable can be explained by the independent variables. In multiple regression models, $R^{2}$ represents the squared correlation between the observed outcomes and the predictor variables used in the model. A higher $R^{2}$ value indicates better model performance.
- RMSE: It measures the average error magnitude in a model’s predictions. It is calculated as the square root of the Mean Squared Error (MSE), quantifying the variance between observed values and the model’s predicted values. A lower RMSE suggests higher model accuracy.
- MAE: Similar to RMSE, MAE evaluates the accuracy of predictions by computing the average absolute difference between observed and predicted values. Its normalized version (nMAE or NMAE) is commonly used for comparison across different scales [26]. Unlike RMSE, MAE is less sensitive to outliers.
- Neurons per layer: The number of neurons in each hidden layer is variable and determined by the optimization algorithm. Each algorithm explores a range of configurations for the two hidden layers, with the number of neurons limited by the search bounds (e.g., between 10 and 100 neurons per layer). Consequently, the architecture provided by each algorithm may vary, optimizing the model based on its specific search process.
- Optimization time: The time taken for optimization will be measured over 50 iterations as each algorithm adjusts the hyperparameters of the model. The total optimization time may vary depending on the algorithm and the complexity of the search space but will be consistently measured across all algorithms.

## 3. Materials and Methods

**Figure 2:**
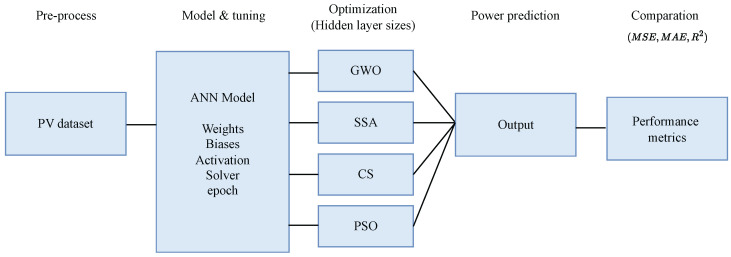
Methodology flowchart.

The proposed methodology shown in Figure 2 for predicting the MPPT begins by pre-processing the PV dataset to ensure it is suitable for model input. An ANN model is then constructed, with key components such as weights, biases, activation function, solver, and epochs tuned for optimal performance. Following this, four optimization algorithms—GWO, SSA, CS, and PSO—are applied to fine-tune the hidden layer sizes and other model parameters. The optimized ANN model generates predictions for MPPT, which are then evaluated using performance metrics: MSE, which measures the average squared difference between predicted and actual values; MAE, which calculates the average absolute difference between the expected and actual values; and the $R^{2}$, which indicates how well the model explains the variance in the actual MPPT values. These metrics assess the accuracy of the model’s predictions, ensuring the effectiveness of the optimization techniques and overall methodology.

### 3.1. Pre-Process

#### 3.1.1. Analysis EDA

The dataset used in this study was obtained from [27]. Four perturbations of different magnitudes were added to the data to simulate partial shade conditions. These perturbations affect both the current and voltage of the photovoltaic panel.

In this analysis, perturbations were explicitly applied to the columns of voltage at the MPP ($V_{mp}\left(V\right)$) and current at the maximum power point (*I_mp(A)*). The perturbation was implemented by multiplying these columns by a reduction factor within certain defined intervals.

#### 3.1.2. Perturbation Equations

For each defined perturbation interval, the Equations (16) and (17) describe the perturbation applied to the $V_{mp}\left(V\right)$ and $I_{mp}\left(A\right)$.

(16) $$V_{mp{\left(V\right)}_{perturbed}}=V_{mp(V)}\times factor$$

(17) $$I_{mp{\left(A\right)}_{perturbed}}=I_{mp(A)}\times factor$$

where:

- $V_{mp(V)}$ is the original voltage value at the maximum power point.
- $I_{mp(A)}$ is the original current value at the maximum power point.
- factor is the reduction factor applicable in the specified interval.

After applying the perturbations, the *Power* column is recalculated using Equation (18).

(18) $$Power_{perturbed}=V_{mp{\left(V\right)}_{perturbed}}\times I_{mp{\left(A\right)}_{perturbed}}$$

The detailed steps followed to apply the perturbations and recalculate the power are described below:

1. The $V_{mp}\left(V\right)$ and $I_{mp}\left(A\right)$ columns are multiplied to create the *Power* column.
2. The perturbation intervals and their reduction factors are defined.
3. The perturbations are applied to $V_{mp}\left(V\right)$ and $I_{mp}\left(A\right)$ within the specified intervals.
4. The *Power* column is recalculated after applying the perturbations.
5. The resulting *DataFrame* is saved to a new Excel file.

**Figure 3:**
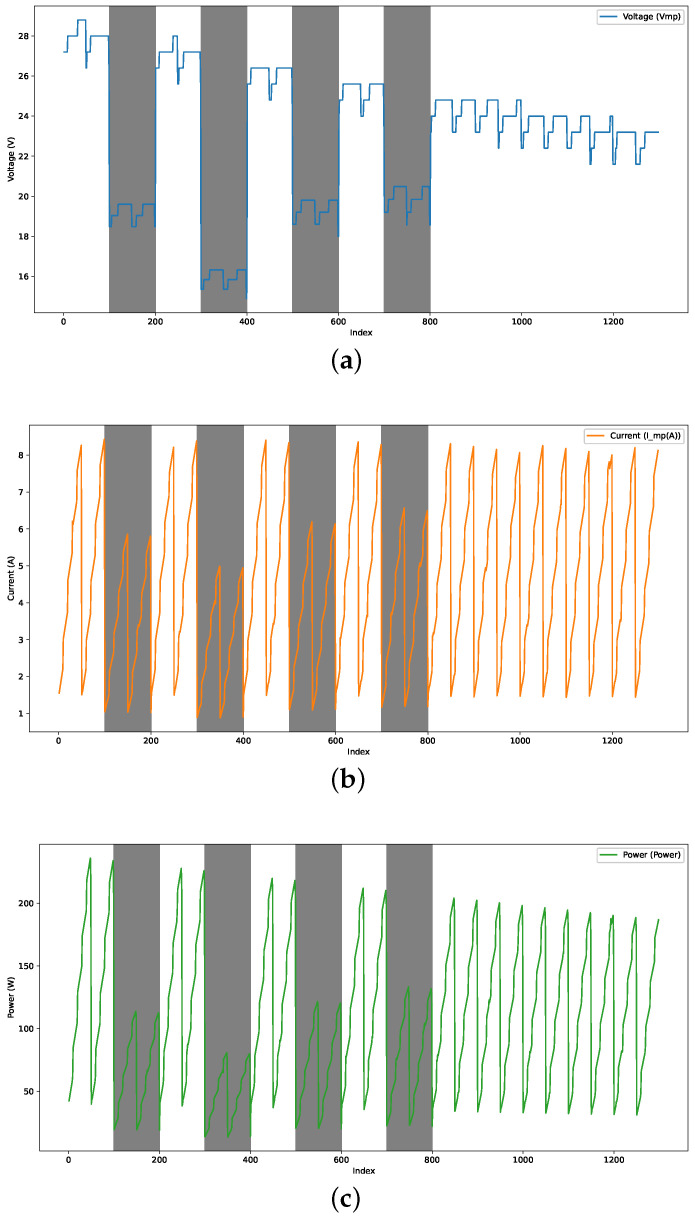
Perturbation results: (**a**) voltage vs. index, (**b**) current vs. index, and (**c**) power vs. index.

As illustrated in Figure 3, these perturbations affect the current and voltage, simulating the partial shade conditions.

**Figure 4:**
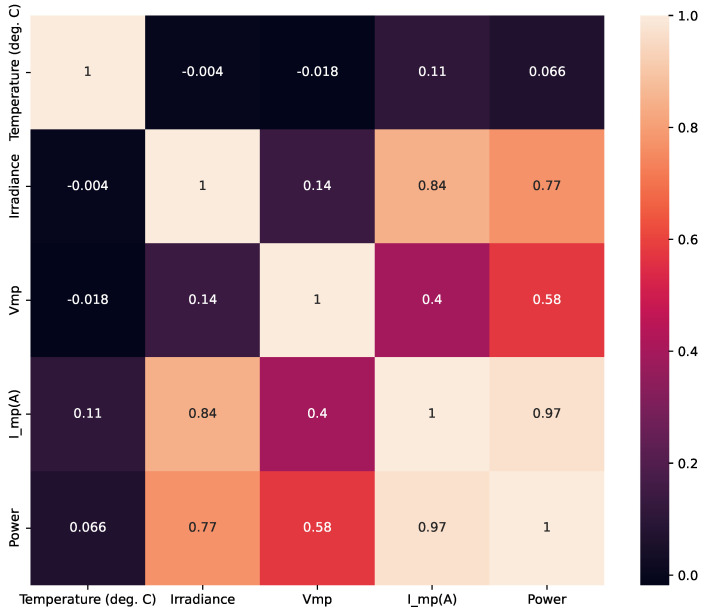
Heatmap of variables.

A correlation matrix shown in Figure 4 was generated to understand the relationships between the variables. A correlation matrix was created with a heat map visualization to observe data trends. It can be observed that the lighter values refer to a direct relationship between the variables. In contrast, a darker value refers to a negative correlation, where one variable increases as another decreases. It is observed that there is no multicollinearity in the data since there are no variables that present a high correlation between the input data. In addition, some trends are identified that directly impact the target variable, which is power:

- Temperature and Voltage (Vmp): There is a strong negative correlation (−0.94), indicating that higher temperatures significantly reduce the voltage.
- Irradiance and Voltage (Vmp): There is a strong positive correlation (0.28), indicating that higher irradiance results in higher voltage.
- Current ($I_{mp(A)}$) and Power: There is a robust positive correlation (0.99), indicating that higher current leads to higher power output.
- Irradiance and Power: There is a strong positive correlation (0.90), suggesting that increased irradiance levels lead to higher power output.

These correlations suggest that Temperature, Irradiance, Current, and Voltage are crucial factors influencing Power.

The theoretical EDA reveals significant relationships between temperature, irradiance, voltage, current, and power. Key observations include:

- Temperature negatively impacts voltage.
- Irradiance is a strong positive influencer of voltage and power.
- Current is a primary power driver with a direct positive correlation.

These insights are critical for understanding the behavior of the photovoltaic system represented by the dataset, and they can inform further analysis and optimization strategies for improving power output under varying environmental conditions.

### 3.2. Model and Tuning

To standardize the input features, the MinMaxScaler from the *scikit-learn* library is employed to scale the data within the range [0, 1].

- Fit the scaler to the input data and then transform the features.

The dataset is divided into training and testing sets to evaluate the ANN’s performance.

- Use the train_test_split function with 80% of the data for training and 20% for testing.
- Set a random state of 42 to ensure reproducibility.

An MLPRegressor from the scikit-learn library is defined to model the relationship between the input features and power output.

- Configure the model with two hidden layers of 64 and 32 neurons, respectively.
- Use the ReLU function as the activation function.
- Employ the Adam optimizer for training, with a maximum of 200 epochs.
- Set a random state of 42 for reproducibility.

The defined ANN model is trained using the training dataset.

- Call the *fit* method with the training data.

The trained model is evaluated on the test dataset to assess its predictive performance.

- Generate predictions for the test set using the predict method.
- Evaluate performance using the following metrics:
  – Mean Squared Error (MSE);
  – Mean Absolute Error (MAE);
  – $R^{2}$ Score.

The evaluation results are printed and visualized to compare the predicted power output against the actual values.

- Create a plot to visualize the actual versus predicted power output.
- Print the performance metrics to the console.

This process ensures that the ANN is accurately trained and evaluated under partial shade conditions, providing a robust model for predicting the power output of a photovoltaic panel.

**Figure 5:**
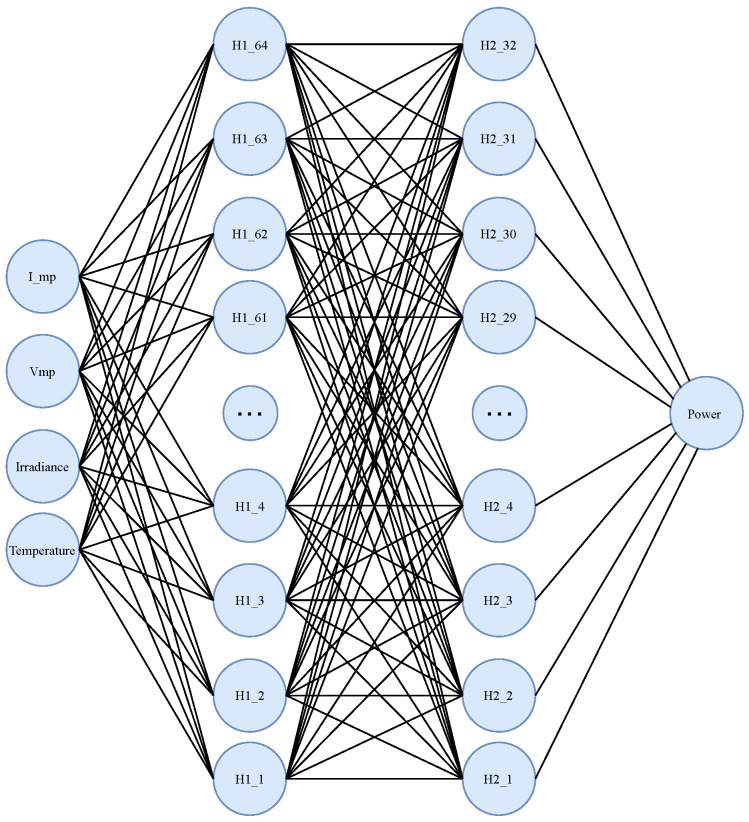
Schematic diagram of the proposed ANN.

The schematic diagram of the ANN used for MPPT in a photovoltaic panel is shown in Figure 5. The diagram shows the input features (Temperature, Irradiance, Vmp, I_mp), the hidden layers with 64 and 32 neurons, and the output layer predicting the power output.

The ANN model was defined with specific hyperparameters to optimize its performance. Unlike model parameters learned during training, hyperparameters must be set before learning begins [28]. Effective hyperparameter optimization enhances model performance by finding the optimal configuration that minimizes error and improves predictive accuracy [29]. The hidden layer sizes were set to 64 and 32 neurons to capture the complex relationships between the input features and the target output. The ReLU activation function was chosen for its effectiveness in deep learning models, helping to mitigate the vanishing gradient problem. The Adam optimizer, known for its adaptive learning rate capabilities, was used to train the model efficiently over 200 epochs. A random state of 42 was set to ensure the reproducibility of the results.

### 3.3. Optimization

Determining the architecture in neural network optimization is critical in achieving optimal performance. One of the primary aspects of optimizing is the number of neurons in each hidden layer, as this directly impacts the model’s ability to learn complex patterns. Bioinspired optimization algorithms can explore a broader search space to facilitate this process. These algorithms are designed to dynamically adjust the number of neurons in each layer, searching for configurations that can enhance the network’s performance by increasing or decreasing the number of neurons per layer. This allows the model to discover the most influential architecture tuned to the specific problem.

Optimizing the size of hidden layers in an ANN is crucial because these layers significantly influence how the network learns to represent and process information. Focusing on hidden layer sizes can be both sufficient and beneficial for several reasons:

- Learning Capacity:
  – Representation Complexity: The sizes of the hidden layers determine the network’s capacity to learn from data by capturing complex features and patterns. More extensive layers enable the network to model more intricate patterns.
  – Balance between Capacity and Generalization: Adjusting the hidden layer sizes allows for finding an optimal balance between a sufficiently complex network to capture functional patterns and one that avoids overfitting by not being overly complex.
- Computational Efficiency:
  – Control of Parameter Count: By modifying the hidden layer sizes, you directly control the number of parameters in the network, impacting memory requirements and training speed, which is crucial in resource-constrained environments.
  – Simplicity in Optimization: Focusing on hidden layer sizes simplifies the optimization process, allowing computational and methodological resources to be concentrated on determining the optimal size without the added complexity of altering other parts of the architecture.
- Impact on Activation Function and Learning:
  – Improvement in Training Dynamics: Adjusting hidden layer sizes can improve the propagation of gradients during training, potentially mitigating issues like vanishing or exploding gradients.
  – Adaptation to Data Characteristics: Depending on the complexity of the data, modifying hidden layer sizes enables the network to better adapt to the specific features of the data being modeled.
- Flexibility and Adaptability:
  – Easy Experimentation: Changing hidden layer sizes is a straightforward and effective way to experiment with different configurations and observe their impact on network performance.
  – Optimization in a Specific Context: If the overall architecture is already effective, optimizing only the hidden layer sizes allows for fine-tuning of the network to meet specific problem requirements without redesigning the entire structure.
- Reduction in Design Complexity:
  – Fewer Design Hypotheses: Concentrating on optimizing hidden layer sizes reduces the number of architectural decisions, streamlining the design process and enabling a more targeted optimization approach.

Optimizing hidden layer sizes is a focused and efficient approach that can lead to significant improvements in the performance of an ANN, particularly when the overall network structure is already well-suited to the task. The goal of the optimization process is to minimize the prediction error of the ANN model. Specifically, the objective function is a combination of the MSE, MAE, and the $R^{2}$ score, which collectively quantify the difference between the actual power output and the predicted power output by the ANN. By minimizing MSE and MAE, and maximizing $R^{2}$, we aim to achieve better predictive performance and a more accurate model.

The optimization problem is mathematically formulated by Equation (19).

(19) $$\min_{h}\;\mathrm{Objective}\left(h\right)=w_{1}\cdot \mathrm{MSE}\left(h\right)+w_{2}\cdot \mathrm{MAE}\left(h\right)-w_{3}\cdot R^{2}\left(h\right)$$

where:

- $h=(h_{1},h_{2},\cdots ,h_{k})$ represents the vector of hyperparameters, specifically, the sizes of the hidden layers in the neural network.
- MSE $\left(h\right)=\frac{1}{n}\sum_{i=1}^{n}{\left(y_{i}-{\hat{y}}_{i}\left(h\right)\right)}^{2}$ is the Mean Squared Error, which penalizes large prediction errors.
- MAE$\left(h\right)=\frac{1}{n}\sum_{i=1}^{n}\left|y_{i}-{\hat{y}}_{i}\left(h\right)\right|$ is the MAE, which provides a more robust measure of error.
- $R^{2}\left(h\right)=1-\frac{\sum_{i=1}^{n}{\left(y_{i}-{\hat{y}}_{i}\left(h\right)\right)}^{2}}{\sum_{i=1}^{n}{\left(y_{i}-\bar{y}\right)}^{2}}$ is the coefficient of determination, which measures the proportion of variance explained by the model.
- $w_{1}$, $w_{2}$, and $w_{3}$ are weights assigned to MSE, MAE, and R², respectively, which control the relative importance of each term in the optimization objective.
- $y_{i}$ is the actual power output for the *i*th sample in the test set.
- ${\hat{y}}_{i}\left(h\right)$ is the predicted power output by the ANN for the *i*th sample, given the hyperparameters h.
- *n* is the total number of samples in the test set.
- $\bar{y}$ is the mean of the actual outputs in the test set.

The optimization algorithm determines the best-hidden layer sizes (number of neurons in each hidden layer) through an iterative process that seeks to minimize MSE and MAE, while maximizing $R^{2}$. The steps are as follows:

1. Initialization:
  - The algorithm generates an initial set of candidate solutions, each representing a different configuration of hidden layer sizes h.
2. Evaluation:
  - For each candidate solution h, the ANN is trained using the training dataset.
  - The performance of the network is evaluated on the test dataset by calculating the combined objective function that includes **MSE**, **MAE**, and $R^{2}$.
3. Selection:
  - The algorithm compares the objective function values for all candidate solutions. The candidate configuration h that results in the lowest objective value is identified as the best-performing configuration in the current iteration.
4. Update:
  - Based on the performance of the current candidates, the algorithm generates a new set of candidate solutions by modifying the hidden layer sizes h.
5. Iteration:
  - Steps 2 through 4 are repeated for a predefined number of iterations or until a stopping criterion is met (e.g., no significant improvement).
6. Convergence:
  - After several iterations, the algorithm converges on a configuration $h^{*}$ that consistently produces the lowest combined error and highest R² score.

At the maximum power point (MPP), the voltage and current values are derived by analyzing the photovoltaic (PV) system’s performance curve. The ANN model is trained to predict these values based on the input dataset, which includes parameters such as irradiance and temperature. The model, after optimization, is able to determine the voltage and current values that correspond to the MPP, ensuring that the system operates at its optimal efficiency.

#### 3.3.1. Particle Swarm Optimization

The PSO algorithm is employed to find the optimal hyperparameters for the ANN, specifically, the sizes of the hidden layers.

- Initialize PSO with 25 particles.
- Define the lower and upper bounds for the hyperparameters as [10, 10] and [100, 100], respectively.
- Set the maximum number of iterations to 100.

*Optimization Process*

The optimization process using PSO involves the following steps:

- Initialization: Randomly initialize the positions of the particles within the defined bounds and set their initial velocities to zero.
- Fitness Evaluation: Evaluate the fitness of each particle using the Mean Squared Error (MSE) of the ANN predictions. The evaluation function trains the ANN with the hidden layer sizes specified by each particle and calculates the MSE on the test set.
- Updating Positions and Velocities: Update the velocities and positions of the particles based on the personal best positions (pbest) and the global best position (gbest) found so far:
  – Update the velocity of each particle using a combination of inertia, cognitive, and social components.
  – Update each particle’s position by adding the updated velocity.
- Exploration and Exploitation: Balance exploration and exploitation by adjusting the inertia weight and using cognitive and social coefficients that guide the search towards personal and global best positions.
- Convergence Tracking: Track the convergence of the MSE, MAE, and $R^{2}$ score over the iterations to monitor the optimization progress.

*Evaluation of the Best Model*

After optimization, the ANN model with the best hyperparameters found by PSO is trained and evaluated.

- Train the ANN with the optimal hidden layer sizes on the training dataset.
- Generate predictions on the test dataset.
- Evaluate the performance using MSE, MAE, and $R^{2}$ score.

The hyperparameters used in this process, including the number of particles, bounds, and activation function, provide a clear overview of the settings optimized by PSO.

#### 3.3.2. Grey Wolf Optimizer

The GWO is employed to find the optimal hyperparameters for the ANN, specifically, the sizes of the hidden layers.

- Initialize GWO with 25 wolves.
- Define the lower and upper bounds for the hyperparameters as [10, 10] and [100, 100], respectively.
- Set the maximum number of iterations to 100.

*Optimization Process*

The optimization process using GWO involves the following steps:

- Initialization: Randomly initialize the positions of the wolves within the defined bounds.
- Fitness Evaluation: Evaluate the fitness of each wolf using the Mean Squared Error (MSE) of the ANN predictions. The evaluation function trains the ANN with the hidden layer sizes specified by each wolf and calculates the MSE on the test set.
- Updating Positions: Update the positions of the wolves based on the positions of the alpha, beta, and delta wolves. The updates are influenced by the best solutions found so far:
  – Calculate the distances from the alpha, beta, and delta wolves.
  – Update each wolf’s position using a combination of these distances.
- Exploration and Exploitation: Balance exploration and exploitation using a linearly decreasing parameter *a*, which influences the update equations.
- Convergence Tracking: Track the convergence of the MSE, MAE, and $R^{2}$ score over the iterations to monitor the optimization progress.

*Evaluation of the Best Model*

After optimization, the ANN model with the best hyperparameters found by GWO is trained and evaluated.

- Train the ANN with the optimal hidden layer sizes on the training dataset.
- Generate predictions on the test dataset.
- Evaluate the performance using MSE, MAE, and $R^{2}$ score.

#### 3.3.3. Squirrel Search Algorithm

The SSA is employed to find the optimal hyperparameters for the ANN, specifically, the sizes of the hidden layers.

- Initialize SSA with 25 squirrels.
- Define the lower and upper bounds for the hyperparameters as [10, 10] and [100, 100], respectively.
- Set the maximum number of iterations to 100.
- Set the flying probability (*p_fly*) to 0.1 and the forgetting probability (*p_forget*) to 0.05.

*Optimization Process*

The optimization process using SSA involves the following steps:

- Initialization: Randomly initialize the positions of the squirrels within the defined bounds.
- Fitness Evaluation: Evaluate the fitness of each squirrel using the Mean Squared Error (MSE) of the ANN predictions. The evaluation function trains the ANN with the hidden layer sizes specified by each squirrel and calculates the MSE on the test set.
- Updating Positions: Update the positions of the squirrels based on their relative fitness:
  – If a squirrel has better fitness than the current best, update the best position and fitness.
  – Squirrels have a probability (*p_fly*) to fly towards the best position.
  – Squirrels that do not fly update their position based on a random combination of their current position and a new random position within the bounds.
  – With a probability (*p_forget*), some squirrels forget their position and move to a new random position within the bounds.
- Convergence Tracking: Track the convergence of the MSE, MAE, and $R^{2}$ score over the iterations to monitor the optimization progress.

*Evaluation of the Best Model*

After optimization, the ANN model with the best hyperparameters found by SSA is trained and evaluated.

- Train the ANN with the optimal hidden layer sizes on the training dataset.
- Generate predictions on the test dataset.
- Evaluate the performance using MSE, MAE, and $R^{2}$ score.

#### 3.3.4. Cuckoo Search

The CS is employed to find the optimal hyperparameters for the ANN, specifically, the sizes of the hidden layers.

- Initialize CS with 25 nests.
- Define the lower and upper bounds for the hyperparameters as [10, 10] and [100, 100], respectively.
- Set the maximum number of iterations to 100.
- Set the probability of abandoning a nest (*pa*) to 0.25.
- Set the step-size parameters (*alpha*) to 0.01 and (*beta*) to 1.5.

*Optimization Process*

The optimization process using CS involves the following steps:

- Initialization: Randomly initialize the positions of the nests within the defined bounds.
- Fitness Evaluation: Evaluate the fitness of each nest using the MSE of the ANN predictions. The evaluation function trains the ANN with the hidden layer sizes specified by each nest and calculates the MSE on the test set.
- Levy Flight: Perform Levy flights to simulate the cuckoo’s random walk, providing a step size for updating the nests.
- Updating Positions: Update the positions of the nests:
  – Compare the fitness of each new nest with the current nest and replace it if the new fitness is better.
  – Abandon a fraction of the worst nests with a probability (*pa*) and generate new nests.
- Convergence Tracking: Track the convergence of the MSE, MAE, and $R^{2}$ score over the iterations to monitor the optimization progress.

*Evaluation of the Best Model*

After optimization, the ANN model with the best hyperparameters (hidden layer sizes) found by CS is trained and evaluated.

- Train the ANN with the optimal hidden layer sizes on the training dataset.
- Generate predictions on the test dataset.
- Evaluate the performance using MSE, MAE, and $R^{2}$ score.

The optimization and model evaluation results are visualized to demonstrate the effectiveness of the CS in optimizing the ANN.

- Plot the MSE, MAE, and $R^{2}$ score convergence over the iterations.
- Plot the actual versus predicted power output to visualize the performance of the optimized ANN.
- Plot the error between the actual and predicted power to highlight any discrepancies.

## 4. Results

The experimental setup can be observed in Table 2. It is observed that the neural network was configured as an experimental base, and subsequently, the optimization models were configured to identify the appropriate number of neurons for the network.

**Figure 6:**
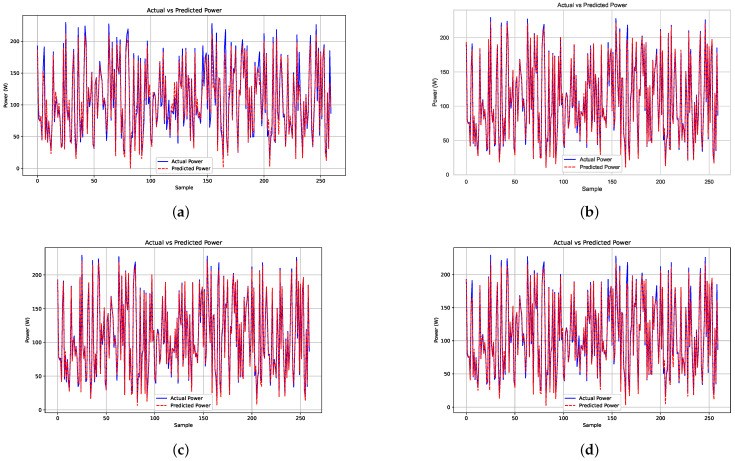

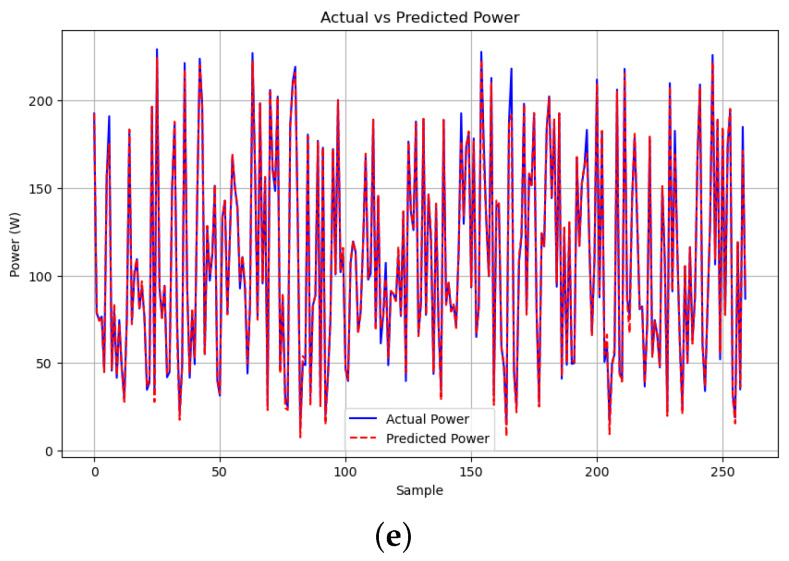
Comparative analysis of actual and predicted power using the ANN and the optimization algorithms: (**a**) ANN, (**b**) GWO, (**c**) SSA, (**d**) CS, and (**e**) PSO.

Figure 6 presents a comparative analysis of the actual and predicted power using different algorithms. Figure 6a shows the prediction made by the ANN, while Figure 6b illustrates the prediction using the GWO. Figure 6c displays the results obtained by the SSA, and Figure 6d shows the prediction made by the CS algorithm. Finally, Figure 6e represents the prediction results obtained using the PSO algorithm.

In the graphs corresponding to the optimization algorithms, the red line, representing the predicted power, follows the blue line, representing the actual power, more closely. This demonstrates the superiority of these algorithms over the ANN in terms of prediction accuracy.

**Figure 7:**
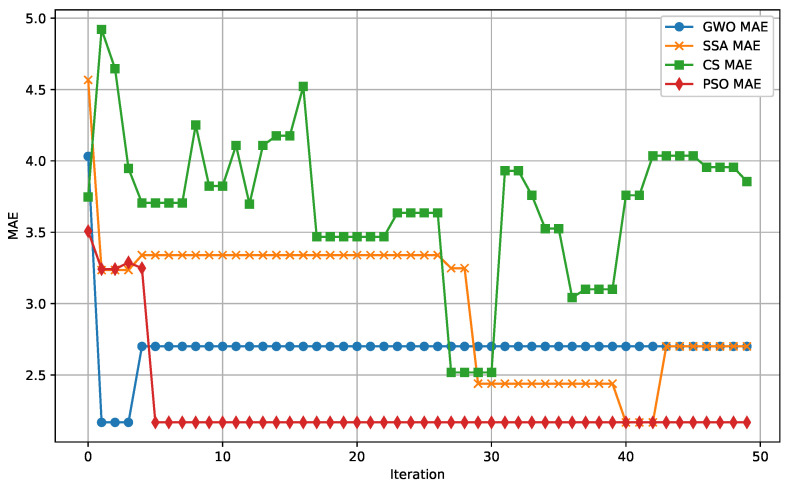
MAE convergence across iterations for all evaluated algorithms. This figure shows the reduction of error as shown by their parameters.

**Figure 8:**
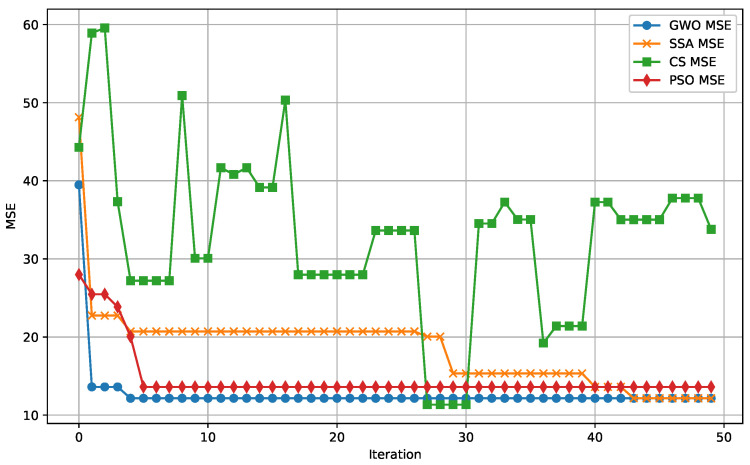
MSE convergence over iterations for all algorithms. This plot demonstrates the rate at which each algorithm minimizes squared errors during training.

**Figure 9:**
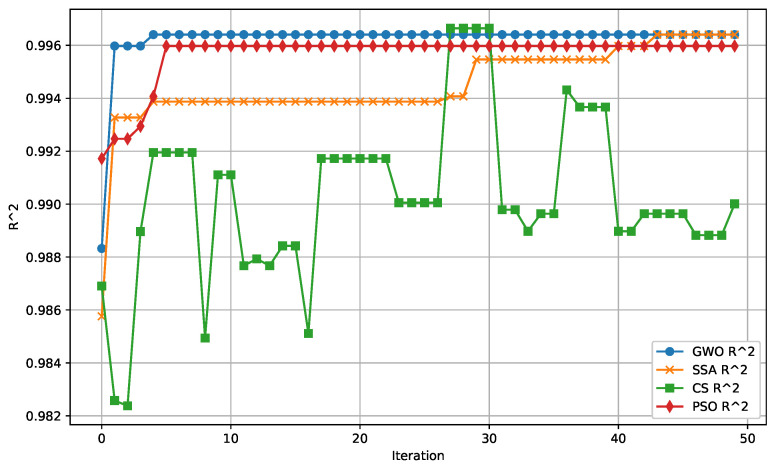
$R^{2}$ score convergence over iterations, indicating the accuracy of the predictions made by each algorithm relative to the actual data. A higher $R^{2}$ score implies a better fit to the data.

**Figure 10:**
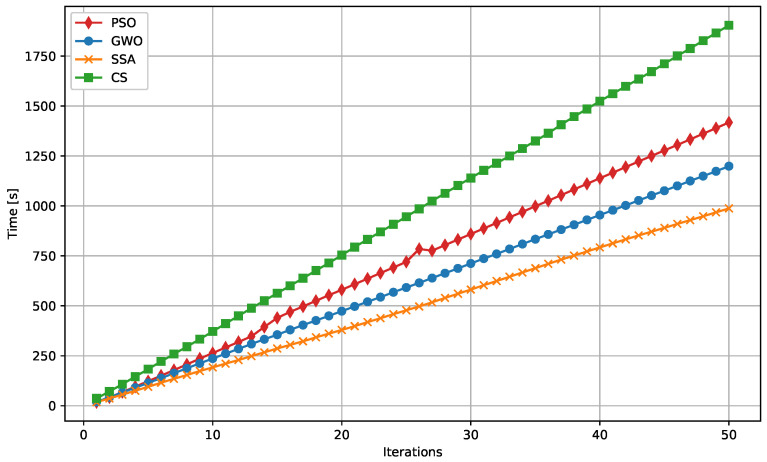
Comparison of computational time vs. iterations for the different algorithms. This figure highlights the efficiency of each algorithm in terms of how quickly they converge to a solution.

The following figures provide a detailed comparison of the performance metrics for the algorithms evaluated. Figure 7 displays the Mean Absolute Error (MAE) convergence for all algorithms, while Figure 8 illustrates the Mean Squared Error (MSE) convergence across iterations. The $R^{2}$ score for prediction accuracy is shown in Figure 9, which highlights how well the models fit the data. Lastly, Figure 10 presents the relationship between computational time and the number of iterations for each algorithm, demonstrating their efficiency.

**Figure 11:**
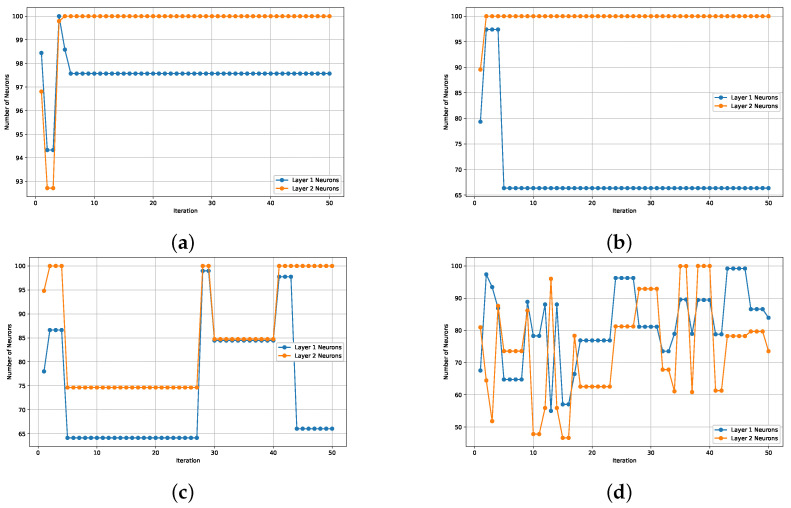
Comparative of hidden layer neuron configurations proposed by the optimization algorithms.

Figure 11 compares hidden layer neuron configurations across optimization algorithms. In Figure 11a, the results for PSO are presented, highlighting the stability of neuron configurations over iterations. Figure 11b illustrates the behavior of the GWO, showing a relatively stable configuration after the initial iterations. Figure 11c corresponds to the Squirrel SSA, where more fluctuation in neuron numbers can be observed, especially in the first layer. Lastly, Figure 11d displays the CS algorithm, which exhibits high variability in neuron configurations across both layers throughout the iterations. This comparison provides insights into the adaptability and stability of each algorithm in determining the optimal number of neurons for the hidden layers.

## 5. Discussion

Table 3 presents a comparison of various algorithms—ANN, PSO, GWO, SSA, and CS—across different performance metrics, such as MSE, MAE, $R^{2}$, execution time, and the number of neurons in each layer of the neural network.

The test performed with a conventional neural network showed prediction evaluation metrics $R^{2}$ of 12.6468, an MSE of 159.9437, and an MAE of 8.0781. This neural network was configured with 64 and 32 neurons for the hidden layers. These values are high compared to the network optimized by PSO, GWO, SSA and CS.

Regarding MSE, GWO achieves the best performance with the lowest MSE value of 11.9487, indicating it has the highest prediction accuracy among the algorithms. SSA and PSO follow, with MSE values of 12.1500 and 13.6064, respectively, showing good accuracy but not as strong as GWO. ANN and CS, with MSE values of 159.9437 and 33.7767, respectively, perform significantly worse, indicating they are less effective in minimizing prediction errors.

For MAE, PSO stands out with the lowest value of 2.1679, suggesting it is the most effective in minimizing the average absolute prediction error. GWO and SSA have slightly higher MAE values of 2.4552 and 2.7003, respectively, indicating good but not the best performance. CS and ANN exhibit higher MAE values of 3.8547 and 8.0781, respectively, implying that their predictions have greater deviations from the actual values.

When considering execution time, SSA is the fastest, completing its tasks in 987.45 s. GWO follows closely with a time of 1198.99 s, while PSO takes 1417.80 s. CS is the slowest, with an execution time of 1904.01 s. ANN does not have a recorded execution time in this table.

Regarding the neural network structure, the number of neurons in each layer appears to influence the performance of the algorithms. For Layer 1, PSO uses 98 neurons, GWO and SSA use 66 neurons, CS uses 84 neurons, and ANN uses 64 neurons. In Layer 2, PSO, GWO, and SSA use 100 neurons, while CS uses 74 neurons and ANN uses 32.

The number of neurons in the hidden layers is critical for determining the model’s capacity to learn complex patterns. PSO, with the most significant number of neurons in both layers (98 in Layer 1 and 100 in Layer 2), demonstrates superior performance in minimizing MAE. This suggests that the more extensive network capacity of PSO allows it to better capture nuances in the data, particularly for reducing the average prediction error. However, this increase in neurons may also contribute to the slightly higher execution time when compared to SSA and GWO.

GWO and SSA, which use 66 neurons in Layer 1 and 100 neurons in Layer 2, show similar performance regarding MSE and execution time. The relatively minor number of neurons in Layer 1 compared to PSO may make these algorithms slightly less accurate regarding MAE, but their performance is still strong overall. The shared architecture of these algorithms highlights that balancing the number of neurons across layers can lead to competitive performance in speed and accuracy.

CS, which uses 84 neurons in Layer 1 and 74 neurons in Layer 2, performs worse than the others in MSE and MAE. This could indicate that the architecture of CS, with fewer neurons in the second layer, may limit its ability to fully capture the complexity of the dataset, leading to higher errors. Similarly, with the fewest neurons in both layers (64 and 32 neurons, respectively), ANN performs worst in both MSE and MAE, likely due to insufficient network capacity.

In summary, GWO emerges as the most balanced algorithm, offering the best performance in terms of MSE and competitive execution time, while using a relatively modest number of neurons in each layer. PSO excels in minimizing MAE, potentially due to its larger network architecture, though this comes at the cost of a slightly longer execution time. SSA, while the fastest algorithm, still provides good accuracy, making it an attractive option for time-sensitive scenarios. ANN and CS, with higher errors and fewer neurons, appear less suited for tasks requiring high precision and speed. This analysis highlights the importance of tuning the number of neurons and the network architecture to optimize performance for specific tasks.

## 6. Conclusions

The comparative analysis of the algorithms—ANN, PSO, GWO, SSA, and CS—under partial shade conditions reveals that GWO remains the most balanced algorithm, excelling in accuracy and computational efficiency. GWO achieves the lowest MSE, indicating superior prediction accuracy, while maintaining a competitive execution time, making it particularly effective in scenarios where the partial shade condition affects the performance of photovoltaic systems. This algorithm strikes an excellent balance, maintaining high accuracy while processing efficiently, which is crucial under fluctuating conditions like partial shading.

Although not as fast as GWO or SSA, PSO shows the best performance in minimizing the MAE, making it a strong contender in environments where reducing absolute errors is crucial, even under partial shade conditions. Its larger neural network structure likely contributes to its ability to minimize prediction errors, though this comes at the cost of longer execution times than GWO and SSA.

While SSA is the fastest algorithm in terms of execution time, it offers a competitive level of accuracy, making it well-suited for time-sensitive applications. Its ability to balance speed with accuracy under partial shade conditions makes it an attractive choice for scenarios where execution time is critical, even though it does not outperform GWO in terms of accuracy.

ANN and CS, on the other hand, exhibit higher errors and slower execution times, making them less suitable for tasks where precision and speed are paramount. This performance gap is particularly noticeable under partial shade conditions, where their ability to maintain accuracy diminishes. Their neural network architectures, with fewer neurons, likely limit their ability to model the complex behavior associated with partial shading in photovoltaic systems.

Overall, GWO stands out as the top-performing algorithm in this comparison, providing an optimal balance between accuracy and execution time, even in the challenging scenario of partial shading. However, the choice of the best algorithm ultimately depends on the specific requirements of the application—whether it prioritizes accuracy, execution time, or a combination of both—especially in the context of partial shade conditions, where performance demands can vary significantly.

In the context of a power prediction model, reducing the error improves the accuracy of predictions, leading to more efficient power forecasting. With the MAE initially at 8.0781 watts, the model’s average predictions were off by that amount. After optimization, the reduced MAE of 2.4552 watts obtained by the GWO reflects a substantial improvement in precision, enhancing power management and minimizing energy waste.

The relevance of a 6-watt difference depends on the application. A 6-watt error can represent a significant percentage of total power output in small systems, such as low-power devices. Although the difference may seem small in larger systems, it can accumulate across multiple units or over time, leading to inefficiencies. In precision-sensitive areas like renewable energy forecasting, even minor improvements can optimize energy usage, reduce costs, and improve overall system efficiency.

In conclusion, the observed relationship between higher power levels and increased error in predictions highlights the inherent non-linearity and complexity of the photovoltaic system, particularly under partial shading conditions. As power levels rise, more significant variations in the input–output relationship make it more difficult for the model to maintain accuracy. This challenge is amplified in fluctuating conditions like partial shading, where prediction errors become more pronounced. By refining the methodology and optimizing the model structure and the number of neurons used, we have demonstrated that algorithms like GWO provide the best balance between accuracy and computational efficiency, particularly in these challenging environments.

Future applications of this methodology could extend to other renewable energy systems, such as wind and hydropower, where similar complexities and fluctuating conditions affect system performance. Integrating hybrid energy systems—combining solar, wind, or other energy sources—could provide further insights into optimizing power generation under varying environmental conditions. Moreover, future research should enhance the optimization algorithms to handle more complex, large-scale datasets and adapt to real-time predictive requirements. These advancements would not only improve the scalability and precision of the models, but also contribute to more efficient energy management and system optimization across a broader range of renewable energy applications, supporting the transition to more sustainable energy systems globally.

## Figures and Tables

**Table 1:** State-of-the-Art Comparison of Optimization Algorithms.

Reference	Optimization Algorithm	Optimized Metric	Precision and Accuracy	Conditions	Tuning
[13]	PSO, GWO, CS, HHO	Power Output, Efficiency	GWO: 98.37%, PSO: 99.32%	Uniform and Non-Uniform Irradiance	PSO: Inertia weight, cognitive and social coefficients
[14]	PSO	Power Output	Efficiency: 93.31%	Partial Shading Conditions	Inertia weight, cognitive and social coefficients
[15]	SHO, PSO	Power Harvesting Efficiency	SHO: 99.81%, PSO: 98.75%	Dynamic Shading, Temperature Variations	PSO: Inertia weight, cognitive and social coefficients
[16]	MPSO, GWO	Power Output, Efficiency	MPSO: 98.42%, GWO: 98.76%	Variable Weather Conditions	MPSO: Inertia weight, cognitive and social coefficients
[17]	SSA	Power Output, Efficiency	Efficiency: 97.65%	Partial Shading, Dynamic Conditions	SSA: Step size, population size
[18]	Firefly Algorithm, EAS	Power Output	Firefly: 97.6%, EAS: 96.8%	Variable Irradiance and Temperature	Firefly: Attractiveness coefficient; EAS: Pheromone update rate
[19]	WOA, ANN, PSO, CS	Power Output, Efficiency	WOA: 98.72%, PSO: 97.55%	Smart Grid Integration	WOA: Spiral updating; ANN: Learning rate

**Table 2:** Hyperparameters setup in the model optimization with PSO, GWO, SSA, and CS.

Hyperparameter	ANN	PSO	GWO	SSA	CS
n	-	25	25	25	25
lb	-	[10, 10]	[10, 10]	[10, 10]	[10, 10]
ub	-	[100, 100]	[100, 100]	[100, 100]	[100, 100]
max_iter	-	50	50	50	50
p_fly	-	-	-	0.1	-
p_forget	-	-	-	0.05	-
pa	-	-	-	-	0.25
alpha	-	-	-	-	0.01
beta	-	-	-	-	1.5
hls	(64, 32)	Variable	Variable	Variable	Variable
activation	ReLU	ReLU	ReLU	ReLU	ReLU
Solver	adam	adam	adam	adam	adam
epochs	200	200	200	200	200
Random state	42	42	42	42	42

n = number of agents, lb = lower bond, ub = upper bond, max_iter = maximum iterations, p_fly = 0.1, p_forget = Forgetting probability, pa = abandonment rate, alpha = step size, beta = flight distribution, hls = hiden layer size, activation = activation function.

**Table 3:** Performance metrics between the optimization algorithms.

Performance Metrics	PSO	GWO	SSA	CS
MSE	13.6064	11.9487	12.1500	33.7767
MAE	2.1679	2.4552	2.7003	3.8547
$R^{2}$	0.99597	0.9964	0.99598	0.9921
No. of neurons (Layer 1)	98	66	66	84
No. of neurons (Layer 2)	100	100	100	74
Execution Time	1417.80 s	1198.99 s	987.45 s	1904.01 s

## Data Availability

Data will be made available upon reasonable request.

## Abbreviations

ANN	Artificial neural network
CS	Cuckoo search
GP	Global peak
GMMP	Global maximum power point [W]
GWO	Grey wolf optimization
IC	Incremental conductance
LMMP	Local maximum power point [W]
MAE	Mean absolute error
MPPT	Maximum power point tracking [W]
MSE	Mean square error [W]
P-V	Power-Voltage [W-V]
P&O	Perturb and Observe
PV	Photovoltaic
SSA	Squirrel search algorithm
PSO	Particle swarm optimization
FOCV	Fractional Open Circuit Voltage
FSCC	Fractional Short Circuit Current
